# Psychological factors and salivary biomarkers (cortisol and opiorphin) in erosive oral lichen planus: A case-control study

**DOI:** 10.34172/joddd.025.43995

**Published:** 2025-12-31

**Authors:** Samira Hajisadeghi, Yasamin Barakian, Robabe Yeganeh, Reza Fotouhi Ardakani, Mohammad Aghaali, Elham Keykha

**Affiliations:** ^1^Research Center for Prevention of Oral and Dental Diseases, School of Dentistry, Baqiyatallah University of Medical Sciences, Tehran, Iran; ^2^Department of Oral and Maxillofacial Medicine, School of Dentistry, Qom University of Medical Sciences, Qom, Iran; ^3^Faculty of Dentistry, Qom University of Medical Sciences, Qom, Iran; ^4^Cellular and Molecular Research Center, Qom University of Medical Sciences, Qom, Iran; ^5^Department of Epidemiology, School of Health, Qom University of Medical Sciences, Qom, Iran

**Keywords:** Anxiety, Cortisol, Depression, Opiorphin, Oral lichen planus, Stress

## Abstract

**Background.:**

Oral lichen planus (OLP) is a persistent inflammatory condition associated with the immune system. Its etiology remains unclear; nevertheless, it seems that individuals with OLP have elevated levels of stress, anxiety, depression, and salivary cortisol. On the other hand, it is expected that the extent of salivary opiorphin changes in the orofacial pain and inflammatory lesions will be high. Moreover, stress, anxiety, depression, salivary cortisol, and salivary opiorphin may affect the perception of pain in OLP patients.

**Methods.:**

This case‒control study included 42 participants: 21 patients with OLP and 21 healthy individuals. The Beck Depression Inventory (BDI), the Perceived Stress Scale (PSS) 14, the Visual Analog Scale (VAS), and the Beck Anxiety Inventory (BAI) were employed to evaluate depression, stress, perceived pain, and anxiety in the case group, respectively. Salivary samples were also collected. Statistical analysis was conducted using SPSS and MEDCALC software at the 0.05 significance level.

**Results.:**

The level of salivary opiorphin was significantly lower in the case group (*P*<0.0001). Also, salivary cortisol in the case group was significantly higher (*P*<0.0001). The correlation between patient age and salivary opiorphin level was significant (*P*=0.03). Salivary opiorphin levels decreased with age. In addition, opiorphin was significantly higher in men than in women (*P*=0.02).

**Conclusion.:**

The present study found that in patients with erosive OLP, salivary opiorphin levels were lower and salivary cortisol levels were higher than in healthy individuals.

## Graphical Abstract

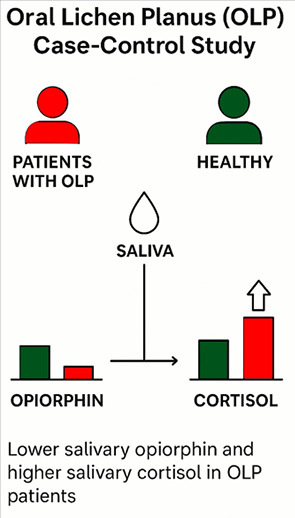

## Introduction

 Oral lichen planus (OLP) is a chronic, immune-mediated inflammatory disease that primarily affects the oral cavity and affects 3‒5% of individuals.^1^ It is most frequently observed in women over the age of 40.^2^ Lichen planus is diagnosed in 50‒70% of patients with oral lesions.^3^ The clinical manifestations of these lesions are reticular, papular, plaque-type, erosive, atrophic, or bullous types.^4^ It is of considerable importance because its ulcerative form is classified as a premalignant lesion.^5^ Although the etiology of this disease remains unclear, it appears that psychological factors, including stress, anxiety, and depression, are significant in its development and progression.^6^ The regulation of immune function is disrupted by stress and other psychological changes, which alter the balance of Th1/Th2 cytokines and increase the Th2 response, which is linked to the development of autoimmune diseases. In patients with OLP, certain studies have demonstrated elevated levels of stress, anxiety, and depression, as well as elevated levels of lesions during the emotional stress period.^1,6,7^ Some researchers have examined the hypothalamic-pituitary-adrenal (HPA) axis by measuring salivary cortisol to clarify the impact of psychological variables on OLP.^1,6,8,9^ Plasma-free cortisol is reflected in salivary cortisol. It is easy and non-invasive to collect.^6^ Stress-induced neuroendocrine hormones may cause immunological dysregulation or change or increase the production of cytokines, which can result in autoimmune disorders.^1^ In stressful situations, the HPA axis is activated, leading to the secretion of cortisol, a hormone that, in addition to influencing inflammatory and immune responses, has a complex effect on the metabolism of carbohydrates, proteins, and lipids. High concentrations of this hormone were observed in individuals with depression, periodontal disease, burning mouth syndrome, and aphthous ulcers.^1^

 Enkephalins, which are classified as endogenous opioids, play an important role in pain perception by binding to opioid receptors and producing the same analgesic effects as morphine. Although enkephalins have a more potent analgesic effect compared to morphine, their analgesic effects do not last as long in terms of two enzymes (neutral endopeptidase and aminopeptidase-N).^10^ Opiorphin is an endogenous pentapeptide that was isolated from human saliva, which has analgesic and antidepressant properties.^11,12^ This hormone was shown to inhibit the enzymes that break down enkephalins, thereby prolonging their effects.^10^ It is highest in tears and saliva. The fact that opiorphin concentrations in tears and saliva are relatively higher than in other body fluids suggests that it may play an important role in the perception of orofacial pain.^10^ Limited studies have been conducted on this subject, and unfortunately, their results are contradictory. Two studies have examined opiorphin levels in individuals with burning mouth syndrome; one indicated elevated opiorphin levels in these patients,^11^ while the other revealed no significant difference.^13^ Central injection of opiorphin was shown to elicit an antidepressant-like effect.^11^ Consequently, it may serve a significant function in painful conditions linked to neurohormonal dysfunction.^14^ Since studies investigating salivary opiorphin in oral lesions are limited, this research aims to evaluate salivary opiorphin levels between patients with OLP and healthy individuals, considering that lichen planus is an inflammatory and painful oral lesion.

## Methods

 This case‒control research received approval from the Ethics Committee of Qom University of Medical Sciences, with ethics ID IR.MUQ.REC.1401.099. The sample size was calculated based on Rödström et al.’s study,^15^ with a mean difference of 1.59 (μ₁ = 6.69, μ₂ = 5.1), standard deviations of 2.2 and 1.4, a significance level (α) of 0.05, and a power (1–β) of 80%. Finally, 21 subjects were included in each group. Also, this study adhered to the STROBE protocol.

###  Inclusion Criteria

 Patients with erosive OLP with symptomatic lesions (whose disease had been confirmed by biopsy), age ≥ 18 years, and not receiving OLP treatment.

###  Exclusion Criteria

 Additional oral mucosal disorders, including burning mouth syndrome, a history of malignant neoplasms, or autoimmune diseases such as lupus erythematosus, rheumatoid arthritis, and Sjögren syndrome, as well as conditions associated with cortisol production like Cushing syndrome and Addison disease, prior use of corticosteroids, oral contraceptives, and medications for xerostomia within the last 30 days, pregnancy, lactation, or hormone replacement therapy for menopause, along with smoking and alcohol dependence.^6^ Taking antibiotics or opioids 48 hours before sampling, decreased salivation in terms of xerostomia and radiotherapy, having dental or craniofacial anomalies, having an infectious disease transmitted through saliva, having chronic or acute pain in the head and neck area due to other causes, or intellectual disability.^10^

 The Beck Anxiety Inventory (BAI)^16^ was employed to evaluate anxiety, while the Beck Depression Inventory (BDI)^17^ was used to measure depression. The BAI questionnaire comprises 21 items, with each item offering four response options (not at all, mild, moderate, and severe) scored from 0 to 3, yielding a maximum total score of 63. Scores are classified as follows: 0 to 9 indicates minimal anxiety, 10 to 16 denotes mild anxiety, 17 to 29 signifies moderate anxiety, and 30 to 63 reflects severe anxiety. A study by Kaviani et al.^18^ demonstrated that the BAI is adequately valid (r = 0.72, *P* < 0.001) and reliable (r = 0.83, *P* < 0.001) in the Iranian population. The BDI also exhibited acceptable reliability (r = 0.73) and good concurrent validity in Iran.^19^ Similar to the previous questionnaire, it has 21 questions with four options that are scored from 0 to 3. The maximum score is 63, and higher scores indicate higher levels of depression. The Perceived Stress Scale (PSS14) was employed to assess stress levels. The PSS, developed by Cohen et al.^20^ in 1983, comprises 14 questions to assess general perceived stress over the past month. It assesses thoughts and feelings regarding stressful events, control, overcoming, coping with psychological distress, and experienced stress. The scale consists of 14 items, each of which has five options (not at all, very little, somewhat, quite a lot, very much), and each option is assigned a score from 0 to 4. The average is calculated for each group, and values that are equal to or greater than the average indicate stress. Pourseyyed et al.^21^ calculated the validity coefficient of this questionnaire at 0.63 and its reliability at 0.73 using Cronbach’s alpha. VAS was used to measure pain intensity, a method often used in clinical research as a valid, simple, and effective way to assess disease control. It ranges on a continuous spectrum, from none to severe pain.^22^ The VAS scale was assessed exclusively in the case group. Before collecting salivary samples, the patients were asked to mark their pain level on a prepared piece of paper. This paper featured a 10-cm line, with the left endpoint labeled “no pain” and the right endpoint labeled “unbearable pain.” The line was ungraduated, with no divisions between the starting and ending points

 The spitting approach was used to gather unstimulated saliva.^23^ Salivary samples were taken at the same time of day to prevent circadian effects. Before saliva collection, the participants were instructed not to consume alcohol for 12 hours, not to eat, drink, chew gum, clean their teeth, or use mouthwash for 90 minutes, and not to engage in any physically demanding activities.^24,25^ Food, lipstick, or blood had to be absent from salivary samples.^23^ An enzyme-linked immunosorbent assay (ELISA) kit was used to determine the levels of opiorphin and cortisol in saliva after collection.

 Statistical analyses were conducted with SPSS and MEDCALC software using t-test, chi-squared test, Pearson’s correlation, and logistic regression tests, and if appropriate, their nonparametric counterparts. All the analyses were deemed to have a significance level of 0.05.^15^

## Results

 Forty-two individuals aged 30‒80 years were assigned to two groups: a case and a control group, with mean ages of 55.67 and 54.57 years, respectively, with only three men in each group. The two groups were matched as much as possible in terms of age and gender. All the subjects were assessed for stress, anxiety, and depression using the PSS14 scale, BAI, and BDI scales, respectively. Patients’ perceived pain was also evaluated using the VAS scale. Salivary samples were collected in both groups.

Table 1 presents the results of the t-test, conducted based on the mean of the variables examined in patients with erosive OLP and healthy individuals. The two groups exhibited statistically significant differences in their salivary cortisol and opiorphin levels.

**Table 1:** Comparison of the means of variables in the patient and control groups

**Variable**	**Number of patients in each group**	**Control group** **Mean±SD**	**Patient group** **Mean±SD**	***P*** ** value**
Stress	21	25.71 ± 7.67	28.57 ± 6.74	0.2
Anxiety	21	11.81 ± 7.08	14.43 ± 12.37	0.4
Depression	21	9.67 ± 8.00	14.52 ± 9.02	0.7
Cortisol	21	8.70 ± 0.13	9.2 ± 0.10	< 0.0001
Opiorphin	21	12.41 ± 1.95	8.58 ± 1.46	< 0.0001

Table 2 illustrates the gender-based comparison of mean salivary cortisol and opiorphin levels between the two groups. The mean cortisol levels were 8.94 in females and 8.99 in males, with no significant difference between the male and female groups (*P* = 0.2). The average opiorphin scores for males and females were 11.94 and 10.25, respectively, with a significant difference between them (*P* = 0.02). Men exhibited a significantly higher level of opiorphin than women.

**Table 2:** Comparison of cortisol and opiorphin in the two gender groups

**Variable**	**Mean±SD for each gender**		***P*** ** value**
	**Male**	**Female**	
Cortisol	8.99 ± 0.04	8.94 ± 0.01	0.27
Opiorphin	11.94 ± 0.66	10.25 ± 0.26	0.02

 To analyze the data obtained from the questionnaires and two salivary factors, SPSS was used. Spearman’s correlation coefficient was calculated for the relationships between each of the variables, and the correlation rate of each of them was obtained. The data were analyzed comparatively in this investigation. A correlation coefficient between 0 and 1 indicated a positive correlation, as the numerical correlation coefficient ranged from 1 to -1. The stronger the positive correlation, the closer the coefficient to 1. In contrast, a correlation coefficient between 0 and -1 indicated a negative correlation, and the closer the coefficient to -1, the more pronounced the negative correlation.

###  Correlation Between Salivary Opiorphin Level and Patients’ VAS

 A positive correlation was observed between the salivary opiorphin levels in the case group and VAS (r = 0.03). However, this correlation was not statistically significant (*P* = 0.8).

###  Correlation Between Salivary Cortisol Level and Patients’ VAS

 There was a negative association (r = -0.06) between the case group’s salivary cortisol levels and VAS. In other words, as one variable’s value increased, the other variable’s value decreased, and vice versa. Nevertheless, there was no statistically significant relationship between salivary cortisol and VAS (*P* = 0.7).

###  Correlation Between Stress, Anxiety, Depression, and Patients’ VAS

 Stress, anxiety, and depression levels in the case group showed positive correlations with VAS scores (r = 0.05, *P* = 0.02, and *P* = 0.09, respectively). In other words, as one variable’s value increased, so did the values of the other variables, and vice versa. Nevertheless, these associations were not statistically significant (*P* = 0.8, *P* = 0.2, and *P* = 0.6, respectively).

###  Correlation Between Opiorphin and Cortisol Levels in Patients’ Saliva

 Salivary cortisol levels in the case group were negatively correlated with salivary opiorphin levels (r = -0.16). In other words, as one variable’s value increased, the other variable’s value decreased, and vice versa. However, this correlation was not statistically significant (*P* = 0.4).

###  Correlation of Opiorphin Level with Stress, Anxiety, and Depression in Patients

 Salivary opiorphin was positively correlated with stress (r = 0.08) and negatively correlated with anxiety and depression (r = -0.02 and -0.06); however, these correlations were not statistically significant (*P* = 0.7, *P* = 0.3, and *P* = 0.7, respectively).

###  Correlation of Cortisol Levels with Stress, Anxiety, and Depression in Patients

 Salivary cortisol levels were positively correlated with these factors (r = 0.1, r = 0.3, and r = 0.05, respectively); however, these correlations were not statistically significant (*P* = 0.5, *P* = 0.1, and *P* = 0.8, respectively).

###  Comparison of the Correlation Between Salivary Opiorphin and Cortisol Levels with Age

 Age in the case group negatively correlated with salivary opiorphin levels. In other words, the salivary opiorphin levels decreased with age. The correlation between patient age and the salivary opiorphin levels was statistically significant (*P* = 0.03). The age of the patient group did not exhibit a statistically significant correlation with salivary cortisol levels (Table 3).

**Table 3:** Relationship between salivary opiorphin and cortisol levels with age in MEDCALC and SPSS software

**Variable**		**Correlation**		***P*** ** value**	
		**opiorphin**	**cortisol**	**opiorphin**	**cortisol**
Age	MedCalc	-0.3	0.1	0.03	0.4
	SPSS	-0.3	0.04	0.03	0.8

###  Comparison of the Correlation Between Stress, Anxiety, and Depression with Age

 No relationship was found between any of the psychological components of stress, anxiety, and depression and age.

###  Comparison of the Correlation Between Stress, Anxiety, and Depression in the case group

 In the case group, anxiety, stress, and depression were positively and significantly correlated (Table 4). These psychological factors did not have a significant relationship with the other variables.

**Table 4:** Correlation between stress, anxiety, and depression in the patient group

**Variable**	**Stress**	**Anxiety**	**Depression**
Stress	-	r = 0.5 *P* = 0.006	r = 0.5 *P* = 0.006
Anxiety	r = 0.5 *P* = 0.006	-	r = 0.4 *P* = 0.02
Depression	r = 0.5 *P* = 0.006	r = 0.4 *P* = 0.02	-

## Discussion

 Research has shown that experimentally increasing the production of pro-enkephalin in the trigeminal nerve ganglion can reduce the pain response to chronic constrictive injury in rats.^26^ Since opiorphin enhances the activation of opioid receptors mediated by enkephalins, and the majority of the enkephalin-driven pain relief in orofacial pain occurs through peripherally located opioid receptors, it is expected that the salivary levels of opiorphin will change in response to orofacial pain.^26,27^ This study included 42 participants: 21 erosive OL patients and 21 controls. Stress, anxiety, depression, salivary cortisol, salivary opiorphin, and VAS were assessed. The results of the study indicated that the mean scores of stress, anxiety, and depression were higher in the case group than in the control group; however, this difference was not statistically significant. In this regard, Girardi et al.’s^1^ research is comparable to ours. The clinical pattern of OLP, inclusion and exclusion criteria, age and gender distribution, and questionnaires employed in their study were similar to those used in our research. Measuring factors such as stress with a questionnaire, despite its reliability and validity, is an objective method, and different conditions and methods may be effective. It is recommended that studies be conducted with a larger sample size and using multiple scales. The present analysis conflicts with the findings of the investigations conducted by Farhad-Molashahi et al.^28^ and Pires et al.^6^ Pires et al. focused on patients with OLP, the majority of whom had non-erosive lichen planus. The average age of our subjects was higher than that of Farhad-Mollashahi and Pires’ studies. Older people may have difficulty understanding the questionnaire questions or may not complete it carefully due to boredom. In the present study, in addition to old age, low literacy levels could have affected the accuracy of completing the questionnaire.

 The case group exhibited a significantly higher mean salivary cortisol level than the control group. Consequently, it can be concluded that there was a statistically significant correlation between salivary cortisol and OLP. The findings of our study are also consistent with those of Waingade et al.,^29^ Koray et al.,^30^ Taghavi et al.,^31^ and Rabiei et al.^32^ However, Pires et al.^6^ and Girardi et al.^1^ did not report a significant difference between the case and control groups. Pires et al.’s^6^ study differed from ours in that the samples were collected from asymptomatic individuals. Cortisol secretion can be influenced by pain and irritation.^33^ Girardi et al.^1^ employed a method (radioimmunoassay) to measure cortisol, which was different from our study. Additionally, our investigation did not identify any statistically significant correlation between subjective stress and salivary cortisol levels. This lack of correlation may be attributed to the participants’ low literacy levels and advanced age, which could compromise the accuracy of questionnaire responses.

 In response to environmental factors such as pain, emotions, stress, and psychological arousal, the peptide opiorphin, which has analgesic and antidepressant properties,^12^ enhances the activity of enkephalins.^13^ According to our research, the case group’s mean salivary opiorphin level was lower than that in the control group. A study by Ruangsri et al.^34^ indicated that reduced levels of opiorphin in unstimulated whole saliva of patients with burning mouth syndrome (BMS) may be linked to the inhibition of the analgesic properties of enkephalin, which can lead to chronic pain.On the other hand, this finding contrasts with the results of a study by Porporatti et al.,^35^ who showed in a meta-analysis study that levels of opiorphin were significantly higher in individuals with orofacial conditions. In their study, high salivary opiorphin levels were observed in chronic, persistent, and acute pain situations, possibly indicating a physiological adaptive response to stress or pain. To date, the role of opiorphin in pain pathways has been controversial. Therefore, further research is crucial to elucidate the mechanistic role of opiorphin in chronic orofacial pain. Additionally, in our study, males had a significantly higher mean salivary opiorphin level than females. In the study by Dufour et al.,^36^ opiorphin levels in saliva and the bloodstream were also reported to be higher in men than in women. They showed that the PROL1 gene, which encodes the precursor of opiorphin, is expressed and translated in various systems of the body, including the reproductive system in males—specifically in the testis, epididymis, and prostate—and in females, in the mammary glands. Additionally, the gene is expressed in the digestive system, particularly in the salivary glands, and in the ocular system, specifically in the lacrimal glands. There was a strong and significant inverse relationship between age and salivary opiorphin in the patient group; in other words, salivary opiorphin levels fall with age. These findings suggest a significant association between salivary opiorphin levels and erosive lichen planus symptoms. Dalirasani et al.^37^ reported no significant difference in opiorphin level between the OLP group and the control group, nor between the two gender groups. However, their study included all types of OLP, so it is not precise enough to generalize to the erosive type. Since opiorphin is an analgesic, we expect it to have a significant change in symptomatic lesions,^10^ and not all forms of OLP are symptomatic. Moreover, the preparation of salivary samples before testing with the ELISA kit should be in a way that inactivates salivary peptidases and removes larger molecules. Otherwise, the amount shown by the kit will not be real.^38^ Using a protease inhibitor cocktail and 0.1% trifluoroacetic acid solution in our study was for this purpose, which was not mentioned in the study by Dalirsani et al.^37^ The group with excruciating oral lesions exhibited higher opiorphin levels, as indicated by the research conducted by Khansari Nejad et al.^39^ It is essential to recognize that their investigation included various painful oral soft tissue lesions in addition to just lichen planus, making it imprecise to apply the findings solely to lichen planus. Additionally, the study by Boucher et al.^13^ confirms our findings by demonstrating an inverse correlation between salivary opiorphin levels and age.

 Our research revealed positive correlations between stress, anxiety, and depression, with no significant relationships with opiorphin, cortisol, VAS, or age. Jafari et al.^40^ demonstrated a positive correlation between anxiety and depression, while cortisol did not exhibit a significant correlation with either of these conditions. On the contrary, Taghavi et al.^31^ showed a relationship between stress and cortisol, and Chaitanya et al.^3^ found a positive correlation of serum cortisol with anxiety and depression.

 To further investigate similar studies on other types of OLP, it is necessary to measure salivary opiorphin in other painful oral and dental lesions, including acute and chronic pain, and measure salivary opiorphin in OLP patients before and after treatment, with larger sample sizes.

## Limitations

 Only patients with painful erosive OLP who had not received any treatment, including topical and systemic treatment, were examined. Due to the fluctuations in cortisol levels throughout the day, sampling needed to be conducted at specific hours. Since most patients were elderly and had low literacy, the researcher carefully explained the questionnaire questions and the VAS. Additionally, to account for the impact of salivary proteases on opiorphin, salivary samples had to be transported to the laboratory under cool conditions after sampling. Autoimmune diseases or other inflammatory lesions can mimic OLP in the oral cavity; therefore, suspected cases were excluded from the study. Furthermore, opiorphin and salivary cortisol testing kits were unavailable in our country and prohibitively expensive.

## Conclusion

 Salivary opiorphin levels were lower in patients with oral erosive OLP, and salivary cortisol levels were higher than in healthy individuals. Also, salivary opiorphin levels were higher in men than in women, and decreased with age in patients.

## Competing Interests

 The authors declare no conflicts of interest.

## Ethical Approval

 This case‒control research received approval from the Ethics Committee of Qom University of Medical Sciences, with ethics ID IR.MUQ.REC.1401.099.
